# Report of Two Cases of Acquired Idiopathic Haemophilia

**DOI:** 10.7759/cureus.20800

**Published:** 2021-12-29

**Authors:** Clara Silva, Mariana Pacheco, João E Silva, Edite Pereira, Jorge S Almeida

**Affiliations:** 1 Internal Medicine, Centro Hospitalar Universitário de São João, Porto, PRT; 2 Internal Medicine, Centro Hospitalar Univeristário de São João, Porto, PRT; 3 Medicine, Faculdade de Medicina da Universidade do Porto, Porto, PRT

**Keywords:** inhibitor, immunosupression, idiopathic, bleeding, factor viii, acquired haemophilia

## Abstract

Acquired haemophilia is a rare haemorrhagic dyscrasia caused by autoantibodies against coagulation factors, most commonly factor VIII (FVIII). Even though about half of the cases are classified as idiopathic, acquired haemophilia is more common in the elderly and/or in individuals diagnosed with other immunogenic conditions such as malignancies, autoimmune diseases, or during puerperium. It can be life-threatening, presenting more frequently with major bleeding.

We report two cases of acquired haemophilia classified as idiopathic in middle-aged patients with no predisposing factors identified during the diagnostic approach: their disease’s progression and complications, choice of treatment, and why and when to change it.

## Introduction

Acquired hemophilia is a rare but serious condition that occurs in individuals who have previously had complete normal haemostasis. The incidence of this disease is one to three cases per million per year, but it is expected to be underreported, and there are not many populational studies in this area [1-5]. Unlike hereditary hemophilia, acquired hemophilia affects men and women equally because it does not involve the genes encoding factor VIII (FVIII, present in the X chromosome), but instead, an inhibitor is produced that binds to said factor and ultimately removes it from the bloodstream. It can virtually affect any clotting factor, with FVIII being the most common target. It has a biphasic distribution, with young women in puerperium and older people around the age of 65 being the most affected. Besides puerperium, there are other risk factors well established, like the presence of an autoimmune disease or a neoplasm. Although its etiology is not entirely understood, it is thought to be related to autoregulatory CD4+ T cells and/or some polymorphisms involving human leucocyte antigen (HLA) or cytotoxic T lymphocyte-associated antigen 4 (CTLA4). The autoantibodies have a non-linear interaction with FVIII (type 2 inactivation kinetics) that starts with a rapid inactivation followed by a slower phase of equilibrium. In this phase, some patients have detectable FVIII levels, but they can be ineffective [1-5].

Patients usually present with cutaneous, mucosal, or muscle bleeding (hemarthroses are relatively uncommon), but the first hemorrhagic manifestation can be massive and fatal. It has a mortality rate ranging between 8% and 28%, depending on the series. It is identified by a prolonged activated partial thromboplastin time (aPTT) with all the other coagulation markers within the normal range, and it can be confirmed by quantifying the factor VIII inhibitor. Taking these facts into consideration, physicians should have a high degree of suspicion towards this entity, enabling an early diagnosis and a prompt and suitable treatment [1-4].

## Case presentation

Case 1

The first patient we include in this report is a 55-year-old male, with a previous medical history of type 2 diabetes mellitus, arterial hypertension, obesity, depression, and Tolosa-Hunt syndrome, diagnosed in 2014, with sequalae amaurosis of the right eye, medicated with prednisolone 10 mg with no recent exacerbation. In August 2017, the patient was admitted to the emergency room for multiple ecchymosis and hematomas of the abdominal wall and upper limbs. During evaluation, a spontaneous uveal haemorrhage was also observed.

Investigation disclosed no cytopenia (normal levels of hemoglobin and platelet counts were documented), and the prothrombin time was normal, at 10.6 seconds. aPTT was very elevated (85.0 seconds), which led to factor VIII dosing with documentation of very low levels (<1%). Inhibitor levels of 132 units of Bethesda (UB) were also documented (Table 1).

**Table 1:** Blood analysis and its changes during treatment UB: units of Bethesda; RTX: rituximab; MMF: mofetil mycophenolate; Pred: prednisolone

Parameter	Aug/2017	Nov/2017 (pred 20mg + MMF)	Feb/2018 (pred 60 mg + cyclophsophamide)	Dec/2018 (pred 20 mg + 1 g RTX 10 months before)	Aug/2020 (pred 5mg + RTX 2g biannually)
aPTT	85.0 sec	105.0 sec	81.0 sec	78.1 sec	40.5 sec
FVIII’s inhibitor titre	132 UB	38 UB	80 UB	62 UB	0,4 UB
FVIII level	<0.01	<0.01	<0.01	<0.01	0,69 (0.70-1.50)

In the face of such findings, it was decided to increase the prednisolone dose to 40 mg/day and to add mycophenolate mofetil (MMF; 750 mg twice a day) to the patient’s therapeutic regimen. He evolved favorably, with a decrease in the inhibitors’ titer and eventual resolution of the haematomas.

Three months later, the patient was again admitted to the ER with worsening haematomas and complaints of ocular pain. A macular haemorrhage in the right eye was observed. He was given recombinant activated VII-factor (90 µg/Kg) and his blood work showed a high inhibitor level for factor VIII (Table 1). Then, his immunosuppressive therapy was altered from 20 mg of prednisolone and MMF to prednisolone 1.5 mg/kg/day plus cyclophosphamide (1 mg/kg/day) for four weeks.

Potentially associated conditions were investigated, particularly other auto-immune diseases. The patient had never had any symptoms suggestive of autoimmune entities, and the markers of auto-immune diseases were all negative. The presence of an occult neoplasm was also pondered and pursued with a thoraco-abdominopelvic CT, upper endoscopy, colonoscopy, and tumoral markers, all of which came back negative. In January and February 2018, the patient had two subsequent admissions for pyelonephritis with concomitant acute kidney injury (AKI). No new haemorrhages or haematomas were apparent, but his FVIII levels were still <1%, with an aPTT of 81 sec and 80 UB of inhibitors (Table 1), while still under high doses of corticosteroids (60 mg/day, up titrated at admission in January).

The patient was therefore started on rituximab 375 mg/m^2^ per week with the intention of a total of four weeks, but he was only administered two doses of the agent as, during the second infusion, he developed an adverse reaction, presenting with a generalized rash. He was kept only on prednisolone from that point on, having been titrated to a minimum effective dose of 20 mg/day. Three months later, in December of 2018, he was readmitted for AKI with nephrotic syndrome. The immunology panel was entirely negative. His acquired haemophilia, with persistently low FVIII levels (<1%), contraindicated the renal biopsy, and the exclusion of concurrent glomerulopathy was, therefore, impossible. As the patient was a long-time diabetic with poor glycaemic control, the introduction and up-titration of angiotensin-converting enzyme inhibitors were performed with recession of proteinuria and frank improvement of renal function.

Despite the stability during the following year (2019), the patient still needed 20 mg of prednisolone to prevent haemorrhagic events and still presented with high levels of inhibitors. As no favorable response was obtained from previous exposure to cyclophosphamide or mofetil mycophenolate, it was decided to restart the patient on rituximab with a slow-rate perfusion. Since the reinstitution of the treatment, he had no further adverse reactions, no hemorrhagic events, and presented only one relevant infection, with no life-threatening complications. The patient is now following a biannual rituximab regime and taking prednisolone 5 mg daily with controlled clinical disease, an aPTT of 40.5 sec, 69% of factor VIII, and 0.4 UB of inhibitors (Table 1).

Case 2

The second case refers to a 45-year-old woman with epilepsy and benign intracranial hypertension, depression, and precocious menopause at the age of 33. The patient was diagnosed with acquired hemophilia in 2003, at 28 years old, during an elective surgery for the correction of hallux valgus. During the procedure, she started bleeding with great difficulty, making it impossible to assess hemostasis, which is only possible after an activated prothrombin complex and activated factor VII transfusions. She had no previous history of recurrent bleeding or hematomas, nor had she undergone any prior surgery. During the evaluation for the blood dyscrasia, she presented a prolonged aPTT, a FVIII deficit with positive inhibitors. She had no history of auto-immune symptoms nor any analytical findings that could suggest an underlying condition. The patient was not pregnant at the time.

She was started on cyclophosphamide at 1.5 mg/kg/day for four weeks and prednisolone at 1 mg/kg/day, with a good response. She then abandoned the follow-up appointments. She resumed follow-up nine years later (in 2013), still on high doses of prednisolone (40 mg/day) and with an aPTT of 64.1 sec, an FVIII title of 0.5% and 8.8 UB of inhibitors. At this time, she presented no relevant spontaneous haemorrhagic events. However, over the following years, in the context of surgical interventions, she required several transfusions and an up-titration of corticosteroids preceding and following such interventions, disclosing disease activity.

In August 2019, it was decided to start the patient on rituximab 375 mg/m^2^ every week for one month biannually. She was re-evaluated six months later, under 20 mg of prednisolone, and had repeated blood work with an aPTT of 53.8 sec and inhibitors of 3.7 UB. Since then, the patient has maintained follow-up and treatment. No haemorrhagic events were reported, nor any relevant infectious complications. Prednisolone was successfully reduced to 5 mg/day, and at her last appointment (April of 2021), she had an aPTT of 42.5 sec and an anti-factor VIII of 2.0 UB.

## Discussion

Acquired hemophilia is a rare condition that can cause fatal bleeding. Almost half of the cases are idiopathic, as is the case with our patients. There are numerous diseases associated with acquired hemophilia, most of which are immune. The first patient that we present has Tolosa-Hunt syndrome. This is a rare condition characterized by recurrent painful ophthalmoplegia with concomitant paresis of the third, fourth, and/or sixth cranial nerves that is caused by granulomatous inflammation. It responds to corticosteroid treatment, but spontaneous remissions may occur. Being a non-specific inflammatory disease, it could be correlated with acquired hemophilia. However, there are no case reports describing the two of them together [2,3,6-8].

The treatment of acquired hemophilia is divided into two phases. In the acute phase, the titer of the inhibitor is of importance in selecting more adequate treatment options to maintain hemostasis. If the titer is inferior to five Bethesda units, a recombinant or concentrate of factor VIII can be given in high doses to overcome the inhibitor. However, if the titer is too high, activated prothrombin complex transfusion should be administered along with activated factor VII, as in such a setting, the usage of factor VIII alone would not be sufficient. After preventing/controlling active haemorrhage, immunosuppression is required to stop the production of inhibitors. Treatment options comprise the use of corticosteroids (prednisolone 1 mg/kg/day), cyclophosphamide (1-2 mg/kg/day for three to six weeks), or rituximab (375 mg/m^2^/week for four weeks), either in monotherapy or in combination. Factor VIII levels should be monitored every two to four weeks to guide treatment [2,3,9]. Due to the rareness of this entity, there is not enough quality evidence to recommend one therapy over the other, and there have even been reports of patients whose factor VIII levels have normalized after the acute phase without any treatment. As previously stated, the goal of treatment is to completely eliminate inhibitors, but it is known that, even if the treatment is successful, the rate at which the titers start to decrease is very slow. In some cases, eradication of the inhibitor cannot be achieved in the desirable period while the bleeding risk remains high. An appreciable percentage of patients present a new haemorrhagic event after beginning the treatment, and up to 20% of patients relapse after a complete remission [2-5].

These patients have had their treatment with corticosteroids and other immunosuppressants prolonged for many months, with no eradication of the inhibitors’ titers and, in the case of the first one, with many infectious complications. From the moment they started the treatment with rituximab, they both managed to reduce the dose of prednisolone and diminish the complications, especially the infectious ones, while having had no more haemorrhagic events. This has allowed for a great improvement in quality of life [2,5].

## Conclusions

We chose these case reports to show an uncommon disease with an even more uncommon set of presentations. Neither of these patients was elderly, nor did they have acquired haemophilia secondary to another immunological disease (either neoplastic or autoimmune). To the best of our knowledge, this was the first case of a patient with both acquired hemophilia and Tolosa-Hunt syndrome. Nonetheless, we could not find any data in our research suggesting these two are related.

Despite being rather rare, this disease, if not recognized and addressed rapidly, can cause life-threatening complications, and we should keep it in our minds when receiving a bleeding patient that has only an altered aPTT, with normal PT on his blood analysis.
